# Moringa Oleifera Seed Extract Concomitantly Supplemented with Chemotherapy Worsens Tumor Progression in Mice with Triple Negative Breast Cancer and Obesity

**DOI:** 10.3390/nu13092923

**Published:** 2021-08-24

**Authors:** Elizabeth R. M. Zunica, Shengping Yang, Ann Coulter, Christy White, John P. Kirwan, Linda A. Gilmore

**Affiliations:** 1 Clinical Oncology and Metabolism, Pennington Biomedical Research Center, Baton Rouge, LA 70808, USA; Elizabeth.Zunica@pbrc.edu; 2 Integrated Physiology and Molecular Medicine Laboratory, Pennington Biomedical Research Center, Baton Rouge, LA 70808, USA; John.Kirwan@pbrc.edu; 3 Department of Nutrition, Case Western Reserve University, Cleveland, OH 44109, USA; 4 Department of Biostatistics, Pennington Biomedical Research Center, Baton Rouge, LA 70808, USA; Shengping.Yang@pbrc.edu; 5 Preclinical Laboratory, Pennington Biomedical Research Center, Baton Rouge, LA 70808, USA; Ann.coulter@pbrc.edu; 6 Office of Executive Director for Basic Science, Pennington Biomedical Research Center, Baton Rouge, LA 70808, USA; Christy.white@pbrc.edu; 7 Department of Clinical Nutrition, University of Texas Southwestern Medical Center, Dallas, TX 75390, USA

**Keywords:** moringa oleifera, triple negative breast cancer, obesity, herbal supplement, chemotherapy

## Abstract

Triple negative breast cancer (TNBC) is an aggressive and highly metastatic breast cancer subtype with limited treatment options. Obesity and insulin resistance are associated with a worse prognosis in those with TNBC. Moringa oleifera (moringa) is a tropical edible plant used for both food and medicinal purposes and found to have anti-obesity and anti-cancer effects in vitro and in preclinical models. The anti-cancer effects of moringa seed extract alone and in combination with chemotherapy were evaluated in immunocompromised female mice with diet-induced obesity bearing MDA-MB-231-derived xenograft tumors. Moringa supplementation protected against high-fat diet- and chemotherapy-induced increases in fasting glucose and improved insulin sensitivity. Moringa supplementation alone did not attenuate tumor growth relative to chemotherapy alone, and in combination worsened tumor progression. Moringa supplementation alone reduced angiogenesis, but this effect was abrogated in combination with chemotherapy. Moringa supplementation may be an effective strategy to improve metabolic health in mice with obesity and TNBC and reduce angiogenesis in tumors, but may have a negative interaction when used as a concurrent complementary therapy. Caution should be taken when considering the consumption of moringa seed extracts while receiving chemotherapy for breast cancer treatment. Further investigations of alternative timings of moringa therapy are warranted.

## 1. Introduction

Cancer is a known comorbidity of obesity, and insulin resistance is associated with an increased risk for breast cancer [1]. Recent studies reported associations between metabolic syndrome and triple negative breast cancer (TNBC), an aggressive and highly metastatic breast cancer subtype with limited treatment options [2,3,4]. Due to the link between obesity, insulin resistance and cancer, several FDA-approved antidiabetic therapies such as insulin sensitizers (thiazolidinediones), insulin secretagogues (sulfonylureas) and biguanides, which have been associated with the decrease in cancer incidence, are being investigated as anti-cancer therapies [5,6,7].

*Moringa oleifera* (moringa) is an edible tropical plant used for both food and medicinal purposes and found to have anti-obesity and antidiabetic effects in rats [8,9] and mice [10,11]. The leaves, bark, flowers, fruit, seeds and root of the moringa plant may be used to make herbal medicines due to the presence of a multitude of bioactive phytochemicals. Moringa leaves are gaining popularity as a supplement and are a rich source of vitamins, essential amino acids and flavonoids, with lower levels of isothiocyanates (ITCs) [12,13]. Moringa seeds have much higher levels of ITCs, particularly enriched in 4-[(α-L-rhamnosyloxy)-benzyl] isothiocyanate, commonly known as glucomoringin-ITC or MIC-1 [10,14]. Multiple studies in C57BL/6J mice with diet-induced obesity have shown that dietary supplementation with either the seed or leaf extract protects against obesity and insulin resistance [10,11].

In addition to improvements in metabolic health, numerous phenolic compounds found in moringa have been shown to act as chemopreventive agents and to have antitumor activity [15,16]. The micronutrient and phytochemical profile and thus the anti-cancer activity of moringa extracts can vary depending on the part of the tree and extraction method used [17,18]. Moringa extracts prepared from leaves induce anti-proliferative effects and induce apoptosis in several cell lines in vitro, including cervical cancer Hela cells [19], human oral epidermoid carcinoma cells [20], MDA-MB-231 TNBC cancer cells [21,22], colon HCT-8 cancer cells [21] and prostate PC3 cancer cells [21,23]. In contrast, a seed extract did not display anti-cancer properties in TNBC and colon cancer cell lines [21]. However, MIC-1-enriched seed extract was found to inhibit the tumor growth of immunocompromised mice bearing ovarian cancer or myeloma cancer tumors [24]. Variability among extracts and anti-cancer outcomes in vitro and in vivo warrants further investigation.

Importantly, the prevalence of herbal medicine use is high in people with cancer, and is commonly used in combination with prescribed antineoplastic treatment despite limited information evaluating herb–drug interaction [25]. Furthermore, women with breast cancer are most likely to use herbal medicines compared to the general population [26,27]. Herbal medicines and nutritional supplements often interact with standard of care anti-cancer treatments including chemotherapy [28,29] and radiation therapy [29]. Therefore, it is critical that anti-cancer investigations of herbal medicines, such as moringa, be in the context of standard of care. Promisingly, moringa leaf extract increases the cytotoxic effect of chemotherapy on pancreatic cells in vitro; however, these observations have not been evaluated in preclinical investigations [30].

Therefore, in this investigation, we evaluated the efficacy of MIC-1-enriched moringa seed extract as a complementary therapy to chemotherapy to inhibit tumor growth compared to chemotherapy alone in an obese xenograft model of TNBC.

## 2. Materials and Methods

Cell Culture. MDA-MB-231 cells were purchased from American Tissue Culture Collection (ATCC, Manassas, VA, USA) and maintained in Leibovitz L-15 medium (Sigma, St. Louis, MO, USA) with 10% fetal bovine serum (FBS) and 1% antibiotic antimycotic at 37 °C, with no CO_2_ supplementation.

Mice. *Rag1^null^* (B6.129s7-Rag1^tm1Mom^/J) female mice were purchased from Jackson Laboratories (Bar Harbor, ME, USA) at 4 weeks of age. Mice were housed 4 per cage in high barrier specific pathogen-free rooms under a 12-h light/dark cycle with *ad libitum* access to water and food. All mouse procedures were performed under strict adherence to protocols approved by the Institute Animal Care and Use Committee at Pennington Biomedical Research Center.

Sixty *Rag1^null^* female mice were fed a high-fat diet (HFD; 60% kcal from fat, D12492). Food intake was recorded weekly per cage. Daily food intake per mouse was estimated as total food consumed per cage/mice per cage × days of food consumption. Individual mouse body weight was measured weekly throughout the study.

After 11 weeks, the mice were subscapularly injected with MDA-MB-231 cells (2.5 × 10^6^ cells in 200 µL of 50:50 Matrigel/Collagen I). Mice were monitored weekly and tumor volume was measured using an electronic caliper, applying the formula [volume = 0.5 × (length) × (width)^2^], where length > width, for approximating the volume of an ellipsoid.

**Figure 1:**
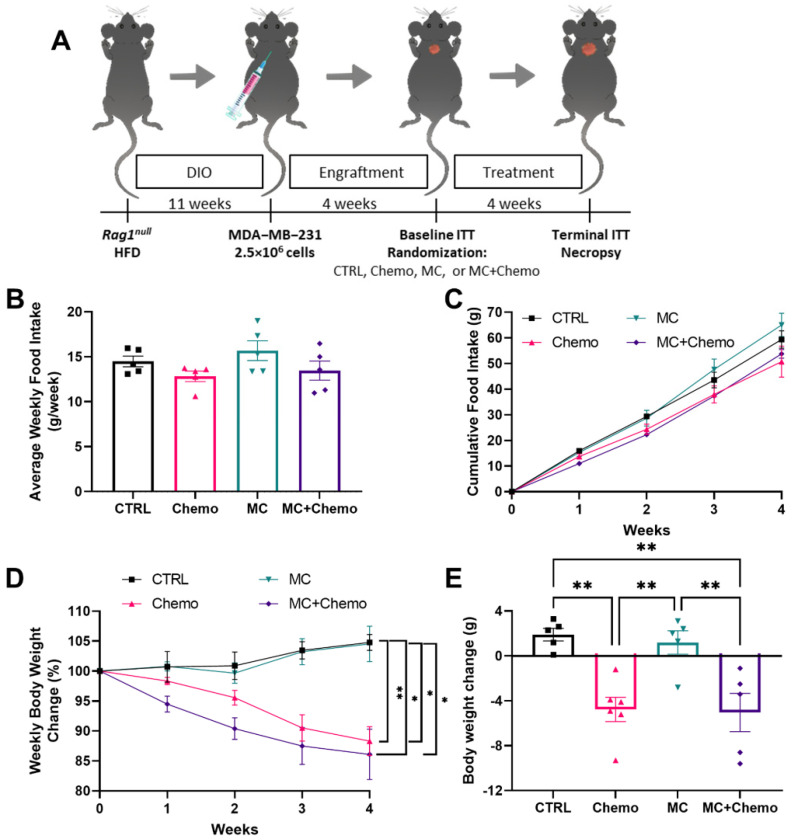
Moringa seed extract concentrate does not alter food intake, body weight, or chemotherapy-induced weight loss in C57BL/6J female mice with diet-induced obesity and TNBC. (**A**) Schematic representations of experimental design. (**B**) Average weekly food intake, (**C**) cumulative food intake, (**D**) weekly body weight change and (**E**) total body weight change (CTRL *n* = 5, Chemo *n* = 6, MC *n* = 5, and MC + Chemo *n* = 5). Data are shown as the mean ± SEM. * *p* < 0.05 and ** *p* < 0.01. Panels A and D were assessed by one-way ANOVA with Tukey’s multiple comparisons. Panels B and C were assessed by a two-way ANOVA with Tukey’s multiple comparisons.

Four weeks after implantation, mice underwent a baseline 120 min. duration intraperitoneal (IP) insulin tolerance test (ITT). Immediately following the ITT, mice bearing tumors ~200 mm^3^ post implantation were randomized for 4 weeks to one of four groups: (1) vehicle control (CTRL; HFD with weekly saline IP injection), (2) chemotherapy (Chemo; HFD with weekly 2 mg/kg doxorubicin and 100 mg/kg cyclophosphamide IP injections), (3) moringa concentrate (MC; HFD supplemented with 0.6% *w*/*w* MC), (4) moringa plus chemotherapy (MC + Chemo; HFD supplemented with 0.6% *w*/*w* MC with weekly 2 mg/kg doxorubicin and 100 mg/kg cyclophosphamide IP injections). After 4 weeks of treatment, mice underwent a post-treatment 120 min. duration IP ITT. Mice were euthanized by CO_2_ inhalation followed by cervical dislocation at study end (Figure 1A).

Cytotoxicity assay. MDA-MB-231 cells were seeded into 96-well plates at 30,000 cells/well and incubated until 90% confluent. After treatment with doxorubicin, cyclophosphamide or MC at various concentrations (0–100 µM, using saline as a 100% viability control and 0.1% Triton X-100 as a negative 0% viability control) for 48 h, the cellular viability was measured by the 3-(4,5-dimethylthiazol-2-yl)-5-(3-carboxymethoxyphenyl)-2-(4-sulfophenyl)-2H-tetrazolium (MTS) assay using CellTiter 96^®^ AQueous Assay kit (Promega, Madison, WI, USA). In brief, a 1:5 dilution of the MTS reagent in complete medium (100 uL/well) was added directly to the adherent cells, and then incubated at 37 °C for 4 h. Absorbance was recorded at 490/630 nm after 90 min using a Cytation 5 Cell Imaging Multi-Mode Reader (Biotek, Winooski, VT, USA). MTS assay data were analyzed using a Non-Linear Fit with variable slope evaluation in Graphpad Prizm^®^ Software, version 8 for Windows. IC_50_ concentrations were calculated using the 2-parameter Hill equation varying between 0 and 100 [31].

Moringa diet. All diets were formulated by Research Diets (New Brunswick, NJ, USA). Moringa concentrate (MC) was obtained from the Botanical Dietary Supplements Research Center. MC from moringa seed extract was prepared as previously reported [32] for the optimization of MIC-1 content. Briefly, moringa seeds were ground and incubated with water at a 1:3 ratio for 2 h at 37 °C. Ethanol was then added to the mixture, which was filtered and dried using a rotary evaporator and freeze-dryer. Dried extract was stored at −20 °C until needed. The concentrate was prepared from multiple batches of extract resulting in 33% MIC-1 by weight. The MC was added to the high-fat diet (D12492; 0.198% MIC-1 (0.6% MC) [11] and was isocaloric to the HFD for fat, protein and carbohydrate content.

Insulin tolerance test (ITT). Mice were weighed and fasted for 4 h (food was removed, cages were changed and mice had access to water). Blood glucose was measured in whole tail-blood after tail nick by glucometer (AccuChek Advantage, Roche, Basel, Switzerland) at 0 (baseline), 15, 30, 60 and 120 min. After baseline blood glucose was measured, human recombinant insulin (0.75 IU/g per body weight, NovolinR, Nordisk, Bagsvaerd, Denmark) was administered via IP injection. ITT data were analyzed and area under the curve (AUC) was calculated for individual mice and averaged for each treatment group.

Hematoxylin and Eosin and CD31 Staining. Tumor sections were collected at necropsy. Tissues were grossed to size and fixed in 10% neutral buffered formalin for 72 h, changing the fixative every 24 h. Tissues were then paraffin-embedded, sectioned to a width of 4 µm and fixed to a glass slide. Slides were then stained for hematoxylin and eosin [33] or CD31. All tumor sections were evaluated and analyzed by a blinded investigator.

For the CD31 staining, formalin-fixed, paraffin-embedded tumor sections were dewaxed, hydrated and incubated in a heat-induced epitope retrieval (HIER) solution (pH 9.0 Tris-EDTA) for 20 min at 100 °C. After cooling, tissue sections were incubated in H_2_O_2_ for 10 min, washed and incubated at 4 °C overnight in CD31/PECAM-1 Antibody (unconjugated; Cat no. AF 3628-SP (R&D Systems, Minneapolis, MN)) diluted 1:500 in Leica BOND primary antibody diluent. Tissue sections were then washed, incubated at room temperature for 15 min in bond polymer (goat probe and goat-on-rodent HRP-polymer), rewashed and counterstained with 3,3’-Diaminobenzidine. Six 20x fields were examined and CD31 staining was analyzed for each region through densitometry quantification using ImageJ software. The percentages of CD31 positively stained area of the six regions were averaged for each tumor.

Total RNA extraction and RNA Sequencing. Snap-frozen tumor tissue pieces were homogenized using a the FastPReop-24^TM^ 5G instrument (MP Biomedicals, Santa Ana, CA, USA) and total RNA was extracted with a miRNAeasy kit (Qiagen Inc, Hilden Germany) per manufacturer’s protocol. RNA was solubilized in RNase-free water. RNA yield and purity were quantified with spectrophotometric absorbance at 230, 260 and 280 nm using a micro volume spectrophotometer (NanoDrop 8000; ThermoFisher, Waltham, MA, USA). Solubilized RNA was stored at −8 °C for downstream applications.

RNA was normalized to 150 ng/µL in nuclease-free water. RNA integrity was assessed using an Agilent Bioanalyzer 2100. Libraries were constructed and sequenced using Lexogen Quant-Seq 3’ mRNA-Seq Library Prep Kit V1.8.8. Briefly, library generation was performed using an oligo(dT) primer, and double-stranded cDNA was purified with magnetic beads. Libraries were amplified using PCR, and transcripts were indexed, pooled and forward-sequenced at 50 bp using Next-seq (Illumina, San Diego, CA, USA). BlueBee software was used to analyze alignment and the DESeq2 V1.24.0 package in R V3.6.1, Rstudio V1.2.1151 and biomaRt V2.40.1 were used for differential expression analysis. Pathway enrichment was analyzed by Ingenuity Pathway Analysis software. Heat maps of differentially expressed genes were visualized by centering the data using z-scores. Z-scores were calculated using the raw gene counts and the following formula: z-score = (LOG_10_(count)-mean)/standard deviation. Transcripts were filtered based on the following criteria: q < 0.05, base mean > 30, and fold change > 1.5. RNA sequencing was deposited in the Gene Expression Omnibus (GEO; Available online: https://www.ncbi.nlm.nih.gov/geo/ accessed on 20 August 2021) repository under the accession number GSE178973.

Quantification and Statistical analysis. Data are reported as mean ± standard error of the mean (SEM) unless otherwise denoted in the figure legend. Statistical analysis was performed with Prism 9 (GraphPad, San Diego, CA, USA). Statistical procedures from individual experiments are detailed in the respective figure legends. Significance was accepted as *p* < 0.05.

## 3. Results

### 3.1. Moringa Seed Extract Concentrate Does Not Alter Food Intake, Body Weight or Total Chemotherapy-Induced Weight Loss in Rag1^null^ Female Mice with Diet-Induced Obesity and TNBC

Food intake was measured weekly and displayed some week-to-week variability, but was not found to be different between the groups. Cumulative food intake was not different between the groups, although after 2 weeks of treatment the MC + Chemo group displayed a decreasing trend (*p* = 0.06) compared to CTRL (Figure 1B). Both the Chemo and MC + Chemo mice displayed overall decreases in body weight compared to CTRL and MC (Figure 1C). Although MC + Chemo did not differ in terminal weight loss compared to Chemo, MC + Chemo displayed a more rapid weight loss early on (Figure 1C). MC + Chemo mice had a decrease in weight compared to MC starting at week 1 and a decrease in weight compared to CTRL starting at week 2 (Figure 1C). Chemo mice had a decrease in weight compared to CTRL and MC starting at week 4 (Figure 1C). Overall, CTRL and MC mice gained body weight, whereas Chemo and MC + Chemo mice lost weight over the duration of treatment (Figure 1D).

### 3.2. Moringa Seed Extract Concentrate Protects against High-Fat Diet- and Chemotherapy-Induced Increases in Fasting Glucose and Improves Insulin Sensitivity in Rag1^null^ Female Mice with Diet-Induced Obesity and TNBC

Just before cancer cell injection, at 16 weeks of age mice received an initial insulin tolerance test (ITT) and all mice displayed a strong decrease in blood glucose 30 min after an insulin injection (Figure S1A). Importantly, the initial AUC was not different between the treatment groups prior to randomization (Figure S1B). Eight weeks after cancer cell injection and just before the start of treatment, all mice underwent a baseline ITT using the same dose of insulin they received 8 weeks earlier and all mice displayed marked resistance to a decrease in blood glucose following the insulin injection (Figure S1C). The baseline AUC also did not differ between groups (Figure S1D).

**Figure 2:**
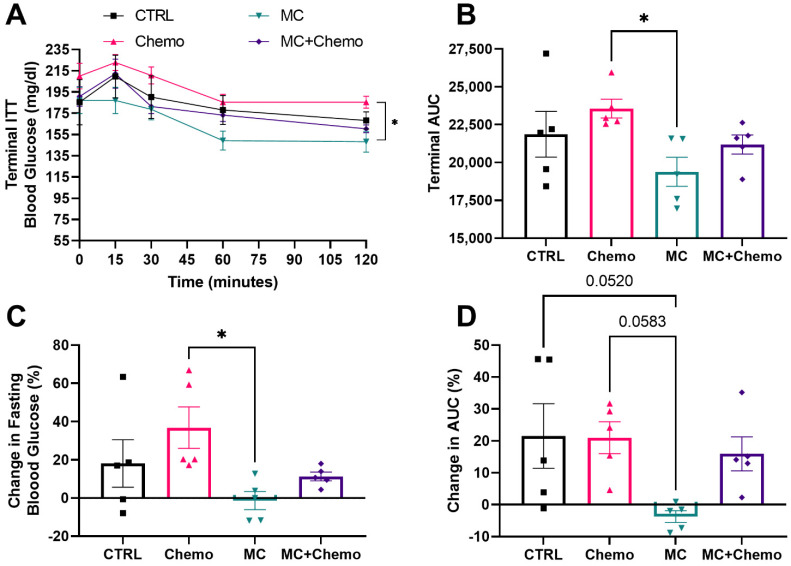
Moringa seed extract concentrate protects against high-fat diet- and chemotherapy-induced increases in fasting glucose and improves insulin sensitivity in C57BL/6J female mice with diet-induced obesity and TNBC. (**A**) Terminal ITT, (**B**) terminal area under glucose response curve (AUC), (**C**) change in fasting blood glucose and (**D**) percent change in glucose AUC (CTRL *n* = 5, Chemo *n* = 6, MC *n* = 5, and MC + Chemo *n* = 5). Data are shown as the mean ± SEM. * *p* < 0.05. Panel (**A**) was assessed by a two-way ANOVA with Tukey’s multiple comparisons. Panels (**B**–**D**) were assessed by one-way ANOVA with Tukey’s multiple comparisons.

After 4 weeks of treatment, a final ITT was performed and MC was found to improve blood glucose response to an insulin injection compared to Chemo (Figure 2A). This resulted in a decrease in the AUC for MC compared to Chemo (Figure 2B). The change in fasting blood glucose from baseline ITT was also decreased in MC compared to Chemo. Strikingly, both Chemo and MC + Chemo resulted in an increase in fasting blood glucose relative to the baseline ITT. CTRL mice also displayed an average increase in fasting blood glucose, but several mice did not display this overall increase (Figure 2C). The change in AUC from baseline was trending towards a decrease in MC compared to both CTRL and Chemo (Figure 2D).

### 3.3. Moringa Seed Extract Concentrate Does Not Reduce Tumor Growth, Has a Negative Interaction with Chemotherapy and Reduces Tumor Angiogenesis in Rag1^null^ Female Mice with Diet-Induced Obesity and TNBC

**Figure 3:**
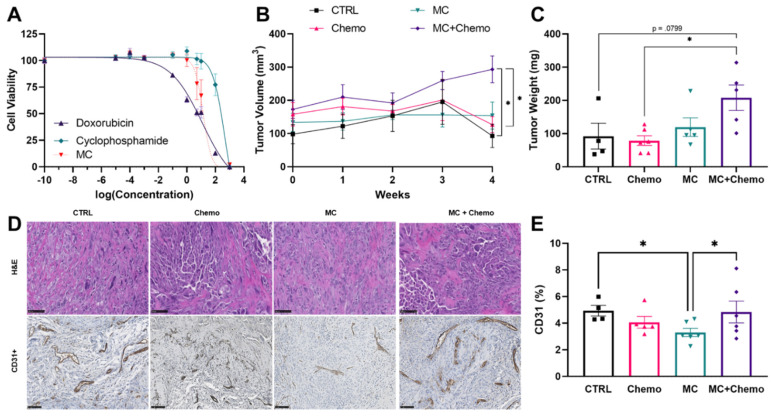
Moringa seed extract concentrate does not reduce tumor growth, has a negative interaction with chemotherapy and reduces tumor angiogenesis in C57BL/6J female mice with diet-induced obesity and TNBC. (**A**) Inhibition of cell viability after 24-h continuous exposure to varying concentrations of doxorubicin, cyclophosphamide or MC in MDA-MB-231 cells (*n* = 10 per condition). (**B**) Tumor volumes over the treatment period and (**C**) terminal tumor mass (CTRL *n* = 5, Chemo *n* = 6, MC *n* = 5, and MC + Chemo *n* = 5). (**D**) Representative H&E (20x, scale = 100 µm), CD31+ (20x, scale = 100 µm) and (**E**) quantification of CD31+. Data are shown as the mean ± SEM. * *p* < 0.05. Panel (**A**) was assessed with a four parameter nonlinear regression. Panel (**B**) was assessed by a mixed-model ANOVA with Tukey’s multiple comparisons. Panels (**C**,**E**) were assessed by one-way ANOVA with Tukey’s multiple comparisons.

Given the efficacy of other moringa extract preparations to inhibit cancer cell growth in several cancer cell lines in vitro, we quantified the IC_50_ of MC to inhibit MDA-MB-231 TNBC cancer cell growth, which was between that of doxorubicin and cyclophosphamide chemotherapies (Figure 3A). We then sought to verify the efficacy of MC to reduce MDA-MB-231 TNBC tumor growth in vivo. We found that MC did not reduce tumor volume compared to other treatment groups and that there was a potential interaction between chemotherapy and moringa such that MC + Chemo mice had larger tumors compared to both CTRL and Chemo (Figure 3B). Similarly MC + Chemo increased tumor weight compared to Chemo and trended towards an increase compared to CTRL (Figure 3C). We performed a histological evaluation of CTRL, MC, Chemo and MC + Chemo tumors and found tumors with chemotherapy (Chemo and MC + Chemo) had large areas of few cells compared to the more densely cellular regions found in the CTRL and MC tumors (Figure 3D). We found that tumors in MC animals had reduced CD31 staining compared to CTRL and MC + Chemo (Figure 3D,E).

### 3.4. Moringa Seed Extract Concentrate Upregulates the Expression of Multiple Genes and Pathways Otherwise Downregulated by Chemotherapy in Tumors from Rag1^null^ Female Mice with Diet-Induced Obesity and TNBC

**Figure 4:**
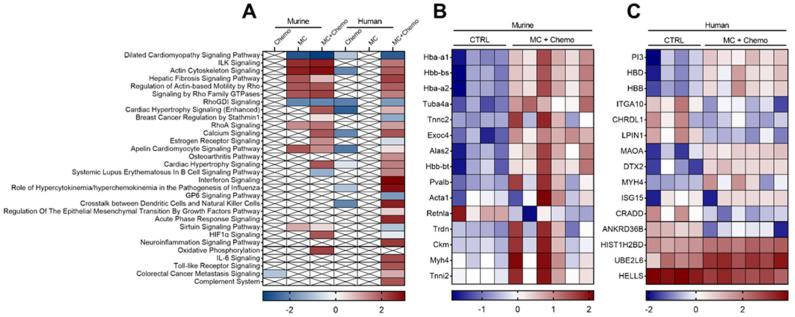
Moringa seed extract concentrate (MC) in combination with chemotherapy (chemo) alters both human and mouse genes in tumors of C57BL/6J female mice with diet-induced obesity and TNBC. (**A**) Heat map visualization of the top 30 dose-responsive canonical signaling pathways following chemo, MC or MC + Chemo treatment evaluated in murine or human genomes. (**B**,**C**) Heat map visualization of top 15 differentially expressed (**B**) murine and (**C**) human genes. Transcripts were filtered based on the following criteria: q < 0.05, base mean > 30 and fold change > 1.5.

To determine if MC altered either the human- and/or murine-derived genes within the tumor, we performed untargeted whole transcriptome sequencing of excised tumors after 4 weeks of treatment. In the murine genome analysis compared to CTRL, we identified 36 transcripts that were differentially regulated by chemo, 74 transcripts that were differentially regulated by MC, and 257 transcripts that were differentially regulated by the MC + Chemo combination therapy. Conversely, in the human genome analysis compared to CTRL, we identified 190 transcripts that were differentially regulated by chemo, 2 transcripts that were differentially regulated by MC and 804 transcripts that were differentially regulated by the MC + Chemo combination therapy. To contextualize these findings, we performed pathway enrichment analysis on the differentially regulated genes in all treatment groups compared to CTRL and then visualized the top 30 canonical signaling pathways (Figure 4A). Interestingly, Chemo did not impact the pathways in the murine genome but did downregulate signaling pathways in the human genome (Figure 4A). In contrast, MC upregulated pathways in the murine genome but did not alter any canonical pathways in the human genome (Figure 4A). Furthermore, the MC + Chemo combination therapy resulted in the most altered pathways in both genomes with a stronger effect on the human genome (Figure 4A). Given the potential interaction between the two drugs in the MC + Chemo combination therapy to worsen both the primary tumor outcome and secondary ITT outcome, we sought to identify the top 15 differentially regulated genes with MC + Chemo treatment (Figure 4B,C). Interestingly, a number of globin transcriptional factors were upregulated in both the murine (Figure 4B) and human (Figure 4C) genomes.

Since MC increased insulin sensitivity in mice and reduced CD31 protein expression in tumors, we sought to evaluate the transcriptional regulation of these pathways in the tumors. In accordance with our overall transcriptome findings, angiogenic and insulin signaling pathways were most changed with the MC + Chemo combination therapy. The combination therapy increased the expression of several *RHO* family genes, *RHOD*, *RHOF*, *RHOJ* and *RHOV* (Figure S1A), and vascular growth genes, *PDGFB*, *PDGFRB*, *Mmp8* and *Mmp24* (Figure S2B), relative to CTRL. With Chemo, we also detected increased expression of some RHO and vascular growth genes, *RHOF* and *Mmp8*, as well as decreased expression of others, *RHOJ*, *Mmp12* and *Mmp13* (Figure S2A,B), relative to CTRL. MC + Chemo increased *SLC2A8* and *Foxo6* genes, while Chemo reduced *IGFBP1* gene expression relative to CTRL (Figure S2C). Additionally, we evaluated several genes in the *CYP/Cyp* family as cytochrome P450 activity is critical for cyclophosphamide activation. MC + Chemo increased *CYP2J2*, *CYP11A1* and *Cyp2e1*, and decreased *CYP20A1*, *CYP26B1* and *CYP51* transcriptional expression relative to CTRL (Figure S2D). Chemo increased *CYP2J2* gene expression relative to CTRL. Taken together, these findings indicate that MC alters the transcriptomic effect of chemotherapy when given in combination with chemotherapy and minimally alters angiogenic and insulin transcriptomic pathways alone.

## 4. Discussion

Cancer is a known comorbidity of obesity, and women with TNBC are more likely to have obesity than normal weight [34]. Obesity is associated with metabolic perturbations including insulin resistance and increased angiogenesis, inflammation and growth factor signaling, resulting in increased tumor initiation and progression [35,36,37]. Additionally, multiple components of cancer treatment such as surgery, chemotherapy and biotherapy induce transient and/or chronic insulin resistance [38,39]. Therefore, pharmaceutical and lifestyle interventions known to attenuate metabolic dysregulation are being investigated as anti-cancer therapies [5,6,7].

Given the previously published efficacy of moringa seed and leaf extract to protect against high-fat diet-induced obesity in male mice, we sought to perform pre-clinical validation of the efficacy of moringa seed extract concentrate to improve metabolic parameters in female mice with high-fat diet-induced obesity and TNBC. Indeed, we observed an improvement in fasting glucose and whole body insulin sensitivity (AUC) with moringa alone compared to chemotherapy, but did not see this effect when moringa was combined with chemotherapy. Much of the anti-obesity effects of moringa were previously observed when moringa leaf or seed extract was administered concurrently with a high-fat or high-fructose diet to lean mice and shown to be protective against diet-induced obesity [11,40,41]. However, in line with Jaja-Chimedza et al. [10], our study demonstrated that moringa seed extract can reduce fasting glucose and improve insulin sensitivity in mice after obesity onset. In addition, these metabolic improvements are not a result of decreased food intake and weight loss as mice receiving moringa alone did not lose weight but mice receiving chemotherapy lost approximately 10% of their body weight during the 4-week treatment period.

In addition to inducing insulin resistance, obesity establishes a pro-angiogenic environment and has been shown to increase vascular growth [37]. Tumor growth and metastasis depend on the vascular network to provide an adequate supply of oxygen and nutrients. The administration of anti-angiogenic compounds has shown efficacy to decrease tumor growth in preclinical models, but displays mixed results in clinical investigations [42]. Part of this discrepancy is attributed to the lack of translational evaluation of the anti-angiogenic compound in combination with standard chemotherapy, and thus there is a critical need to expand preclinical investigation to include combination therapy [43,44]. Promisingly, moringa leaf extract has demonstrated anti-angiogenic properties and has been shown to inhibit retinal angiogenesis in streptozotocin-induced diabetic rats [45], and it downregulates the transcriptional expression of *VEGF*, VEGF receptor 2 (*KDR*) and endothelin-1 (*End-1*) in the cervix of preterm labor mice models [46]. To date, the anti-angiogenic properties of any preparation of moringa have not yet been evaluated in tumors. With moringa seed extract, we observed decreased protein expression of the prognostic angiogenic marker, CD31, in the tumors. Importantly, moringa did not reduce tumor angiogenesis in combination with chemotherapy.

We found that the combination therapy prevented the anti-angiogenic potential of the moringa seed extract and resulted in the transcriptional upregulation of key pro-angiogenic genes. Notably, hypoxia inducible factor (*HIF1α*) signaling, a critical activator of angiogenesis, was transcriptionally upregulated in the moringa chemotherapy combination-treated tumors, but not with moringa alone. Additionally, the combination treatment upregulated Rho family GTPase signaling, including *RhoA* and *VEGF*-driven actin-based motility genes, which play an important role in endothelial cell organization and neoangiogenesis [47,48,49]. Furthermore, moringa with chemotherapy transcriptionally increased the expression of multiple matrix metalloproteinases (*MMPs*), which transcribe proteolytic enzymes critical for vascular remodeling and angiogenesis enhancement [50].

Although we observed an improvement in fasting glucose and whole body insulin sensitivity and a reduction in angiogenesis with moringa alone, moringa seed extract did not reduce tumor volume in mice with diet-induced obesity compared to other treatment groups. Moringa has consistently been shown to reduce fasting blood glucose and improve glucose tolerance in obese and diabetic preclinical and clinical studies. However, evidence regarding the effect of moringa on insulin levels is not as robust, with some studies demonstrating an increase in circulating insulin [51]. Hyperinsulinemia is a well-known driver of tumor growth and progression [52], and thus future investigations to measure circulating insulin as well as tissue-specific changes in insulin sensitivity in response to moringa are warranted. Strikingly, mice that received the moringa and chemotherapy combination therapy had larger tumors compared to both control and chemotherapy alone. The combination treatment transcriptionally upregulated tumor expression of *SLC2A8* and *FoxO6*, important genes for glucose uptake [53], metabolism [54] and redox balance [55]. Our study demonstrates that moringa and chemotherapy did not have an additive benefit to attenuate tumor growth. In fact, we observed an herb–drug interaction between moringa and chemotherapy.

The interaction between herbal and dietary supplements and chemotherapy is not uncommon [56,57]. Many herbal supplements are capable of altering physiological processes, potentially decreasing chemotherapy efficacy and/or medication toxicity [28]. However, data regarding the combination of ITCs and chemotherapy are limited [30]. We identified several genes and pathways known for promoting cancer cell proliferation that were downregulated by chemotherapy and conversely upregulated by the moringa chemotherapy combination. The combination therapy increased integrin-linked kinase (*ILK*) signaling [58] and genes responsible for coding hemoglobin subunits. Orthotopically implanted MDA-MB-231 cells overexpressing hemoglobin subunit beta (*HBB*) demonstrated increased tumor growth and neoangiogenesis [59]. In addition, the upregulation of *HBB* promotes epithelial–mesenchymal transition and has been commonly observed in circulating tumor cells of breast, prostate and non-small cell lung cancer patient-derived xenograft models [60]. Together, these transcriptional changes support potential mechanisms for future investigation that may be the result of moringa chemotherapy interactions contributing to increased tumor growth with combination therapy.

In addition, cyclophosphamide requires biotransformation by cytochrome P450 (CYP) enzymes to be an effective cytotoxic alkylating agent [61]. Many drug–herb interactions are thought to result from the inhibitory effect on these CYP enzymes blocking the activation of cyclophosphamide [61]. Indeed, moringa extracts have been shown to inhibit liver *CYP* isozymes including *CYP1A2* [62], *CYP3A4* and *CYP2D6* [63]. However, CYP enzymes constitute a superfamily of more than 50 isoforms responsible for both activating and detoxifying drugs. While hepatic CYP2B6 and CYP2C19 have the highest activation of cyclophosphamide, additional isoforms including CYP2C9, CYP3A4, CYP3A5 and CYP2J2 can also activate the prodrug [64]. Therefore, inhibiting some but not all isoforms may not decrease the effectiveness of cyclophosphamide [65]. CYP isozymes are also expressed in extrahepatic tissues [66] and cancers [67], including MDA-MB-231 cells [68]. We found that moringa alone did not alter the transcription of the CYP isozymes detected in the MDA-MB-231 tumors. The transcription of *CYP2J2*, known to metabolize cyclophosphamide [64], was upregulated in the tumors by chemotherapy alone and by combination therapy. This upregulation of *CYP2J2* is not surprising as exposure to cyclophosphamide can increase the transcription and expression of cyclophosphamide metabolizing enzymes [69]. Ultimately, more research is needed to truly understand the complex mechanisms involved in herb–drug interactions and their impact on clinical outcomes.

These results are taken within the context of the limitations of this study. Further investigation of the phenotypic and mechanistic interaction between cytotoxic therapies used in the clinic and moringa oleifera extracts is critical as the global market for moringa products is estimated to reach USD 8.4 billion by the year 2026 [70]. Additionally, phytochemical distribution varies within different parts of the plant and various processing techniques can influence the extraction, and thus the examination of well-characterized extract preparations is warranted [18,32]. Here, we investigated the effects of a single batch of moringa seed extract on the growth of established tumors in xenografts. We are unable to extrapolate our findings to other cancer types, treatment modalities and time-points in the cancer control continuum. Similar to the protection against diet-induced obesity, moringa may have a role in chemoprevention. While more research is needed before evidence-based recommendations can be given, we caution patients about consuming moringa oleifera products while receiving chemotherapy for breast cancer treatment.

## Data Availability

The RNA sequencing datasets produced in this study are available at Gene Expression Omnibus (GEO) under the accession number GSE178973.

## Supplementary Materials

The following are available online at https://www.mdpi.com/article/10.3390/nu13092923/s1, Figure S1: Related to Figure 2, Figure S2: Related to Figure 4.
