# Supplementary material for: Moringa Oleifera Seed Extract Concomitantly Supplemented with Chemotherapy Worsens Tumor Progression in Mice with Triple Negative Breast Cancer and Obesity
Source: Nutrients. 2021 Aug 24;13(9):2923. doi: 10.3390/nu13092923 (PMC8472177; doi:10.3390/nu13092923)
Supplement: Supplementary file 1 [file nutrients-13-02923-s001.zip › nutrients-1314325-supplementary.pdf]

Supplementary Materials: The following are available online at www.mdpi.com/xxx/s1.

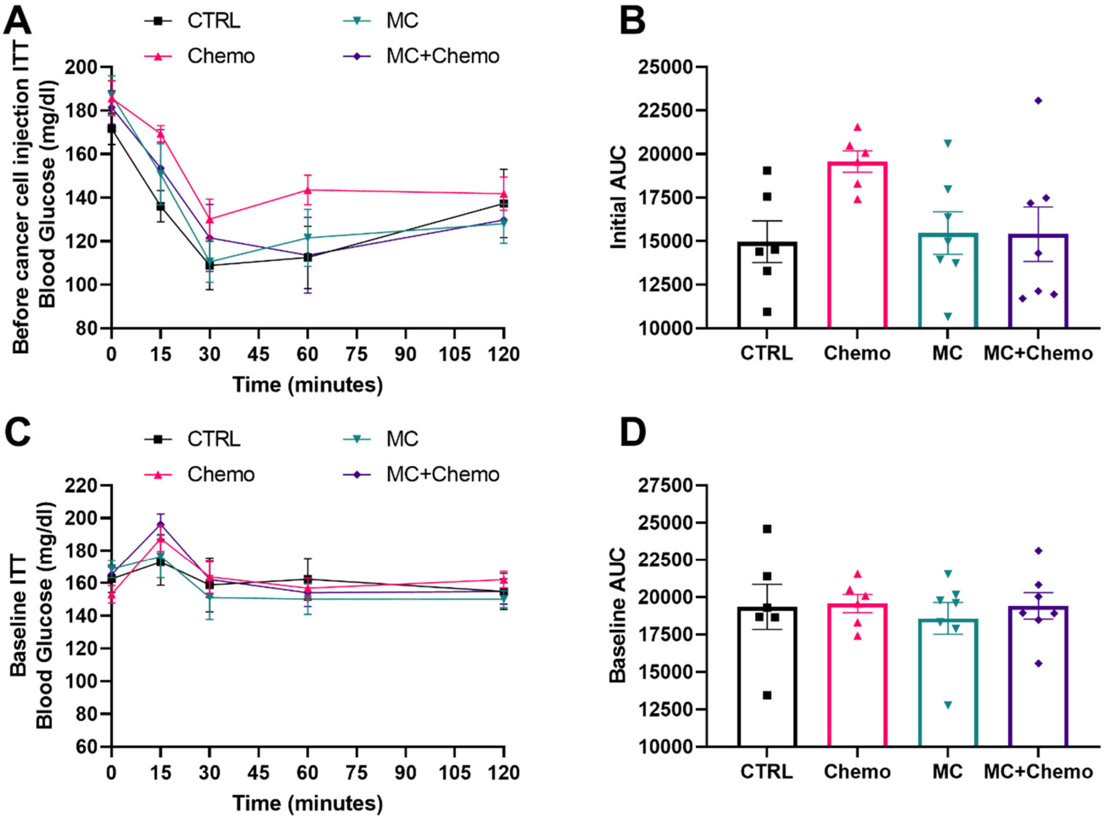

Figure S1. Related to Figure 2.

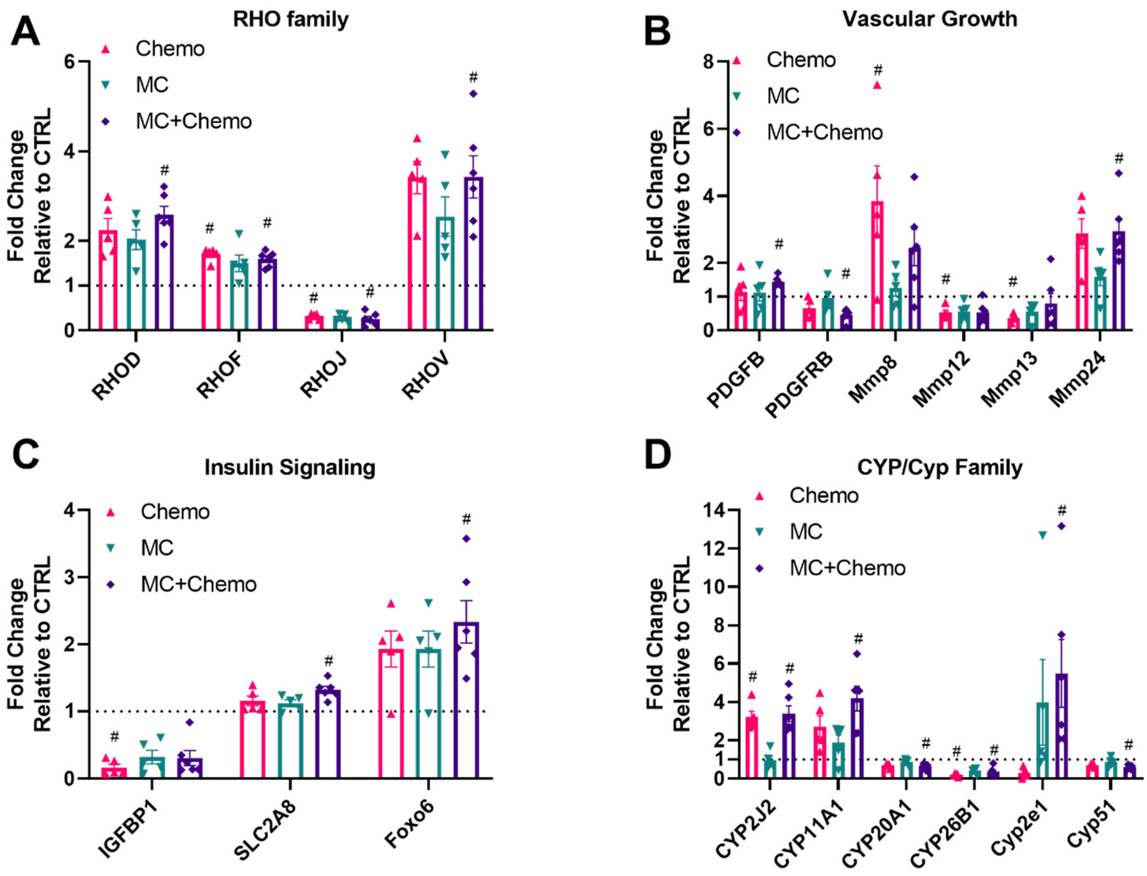

Figure S2. Related to Figure 4. Data are shown as the mean ± SEM. # padj<0.15
